# Inhibition of extracellular matrix mediated TGF-β signalling suppresses endometrial cancer metastasis

**DOI:** 10.18632/oncotarget.18069

**Published:** 2017-05-22

**Authors:** Subhransu S. Sahoo, Min Yuan Quah, Sarah Nielsen, Joshua Atkins, Gough G. Au, Murray J. Cairns, Pravin Nahar, Janine M. Lombard, Pradeep S. Tanwar

**Affiliations:** ^1^ Gynaecology Oncology Group, School of Biomedical Sciences and Pharmacy, University of Newcastle, Callaghan, New South Wales, Australia; ^2^ The Picornaviral Research Unit, School of Biomedical Sciences and Pharmacy, University of Newcastle, Callaghan, New South Wales, Australia; ^3^ Hunter Cancer Biobank, University of Newcastle, Callaghan, New South Wales, Australia; ^4^ Discipline of Pharmacy and Experimental Pharmacology, School of Biomedical Sciences and Pharmacy, University of Newcastle, Callaghan, New South Wales, Australia; ^5^ Department of Maternity and Gynaecology, John Hunter Hospital, New Lambton Heights, New South Wales, Australia; ^6^ Department of Medical Oncology, Calvary Mater Newcastle, Waratah, New South Wales, Australia

**Keywords:** microenvironment, ECM, endometrial cancer, TGF-β signalling, metastasis

## Abstract

Although aggressive invasion and distant metastases are an important cause of morbidity and mortality in patients with endometrial cancer (EC), the requisite events determining this propensity are currently unknown. Using organotypic three-dimensional culture of endometrial cancer cell lines, we demonstrated anti-correlated TGF-β signalling gene expression patterns that arise among extracellular matrix (ECM)-attached cells. TGF-β pathway seemed to be active in EC cells forming non-glandular colonies in 3D-matrix but weaker in glandular colonies. Functionally we found that out of several ECM proteins, fibronectin relatively promotes Smad phosphorylation suggesting a potential role in regulating TGF-β signalling in non-glandular colonies. Importantly, alteration of TGF-β pathway induced EMT and MET in both type of colonies through slug protein. The results exemplify a crucial role of TGF-β pathway during EC metastasis in human patients and inhibition of the pathway in a murine model impaired tumour cell invasion and metastasis depicting an attractive target for therapeutic intervention of malignant tumour progression. These findings provide key insights into the role of ECM-derived TGF-β signalling to promote endometrial cancer metastasis and offer an avenue for therapeutic targeting of microenvironment derived signals along with tumour cells.

## INTRODUCTION

The extracellular matrix (ECM) is a major component of the cellular microenvironment and regulates normal tissue development and homoeostasis. Stromal-epithelial communication in early development and steroid signalling is important for normal uterine functions. ECM of female reproductive tract undergoes extensive structural remodelling for decidualization, implantation and endometrial regeneration [1]. In contrast, abnormal ECM dynamics contributes to the pathological processes such as endometriosis, infertility, cancer and metastasis. The signalling alteration in uterine stroma or ECM that regulates remodelling of the differentiated endometrium to a disease or metastatic cancerous state is currently unclear. Studies have shown the crucial role of stromal signals in controlling the proliferative potential of endometrial epithelium [2–4]. Epi-genome wide methylation analysis revealed hypermethylation of Hand2 gene in endometrial stroma significantly contributes to endometrial cancer [5]. Adipocytes in the microenvironment of higher body mass index (BMI) patients also promote endometrial cancer development [6, 7]. Given that stromal components probably significantly contribute to growth and development of endometrial cancer, relatively limited work has been carried out to address the role of the ECM in uterine biology and cancer.

The tissue microenvironment comprises of fibroblasts, adipocytes, immune cells as cellular and ECM as a non-cellular component and is known for its role in maintaining the integrity of normal tissue architecture. Disruption of the normal balance between epithelial cells and the surrounding stroma leads to tumour progression [8, 9]. These early neoplastic changes are highly reflected by the ability of tumour cells to misdirect the surrounding stroma and turn it from restrictive to the supportive environment. Once the tug of war is won by tumour cells, the restrictive microenvironment itself sustains the tumour cells to disseminate. This suggests that progression of occult tumours to frank carcinomas require significant changes in the microenvironment [10]. Thus, within the mature tissue, cell autonomous heterogeneity due to the mutations in oncogene or tumour suppressor gene is not sufficient to cause cancer unless the molecular signalling cascade has been perturbed by the microenvironment.

Cellular heterogeneity cannot be exclusively interpreted by random biological noise but there are substantial contributions from a cell's local surrounding environment [11, 12]. As compared to conventional two-dimensional (2D) cell culture, organotypic three-dimensional (3D) basement membrane cultures allow monitoring of cell-to-cell differences by providing an extra dimension to grow and by supporting cells in reconstituted basement membrane matrix or ECM [13]. The more realistic geometry of *in vivo* organisation and ECM context can give rise to non-genetic variations of a cell at its molecular level [14].

3D cell culture models have been widely used in many epithelial cancers to study cellular phenotypic changes and drug resistance mechanism [15–18]. In this study, we have addressed the molecular alterations of a cell by changing its environment and determined correlation of phenotypic divergence to the propensity of cancer progression. Using 3D basement membrane cultures of human uterine epithelium (endometrium) originated cancer cells, we have uncovered a dynamic heterogeneity that develops consecutively from 2D to 3D culture in absence and presence of ECM. ECM attached endometrial cancer cells form distinct glandular and non-glandular architecture. The dynamic molecular cascade regulating this discrete phenotype is controlled by anti-correlated transcriptional programs of the transforming growth factor-β (TGF-β) signalling pathway. The dichotomous role of TGF-β signalling as pro-tumorigenic or tumour suppressive is well known in many human cancers [19]. Cancer cells either avoid the tumour suppressive action of TGF-β through inactivation of membrane receptors or undergo a TGF-β induced epithelial-mesenchymal transition (EMT) that promotes cancer cell invasion and metastasis [20]. Here we show that the TGF-β pathway is upregulated in ECM attached cells not forming glands whereas the same signalling is downregulated in cells forming glands. The cellular heterogeneity adapted due to the matrix is also reversed by either activation or suppression of TGF-β signalling. On the other hand, the cellular phenotypic and molecular changes strongly correlate with the metastatic feature of cancer cells. These observations have very significant implications with respect to examining adaptive cellular heterogeneity due to microenvironment and its impact on cancer metastasis.

## RESULTS

### EC cells have distinct phenotypic divergence in different microenvironment

**Figure 1:**
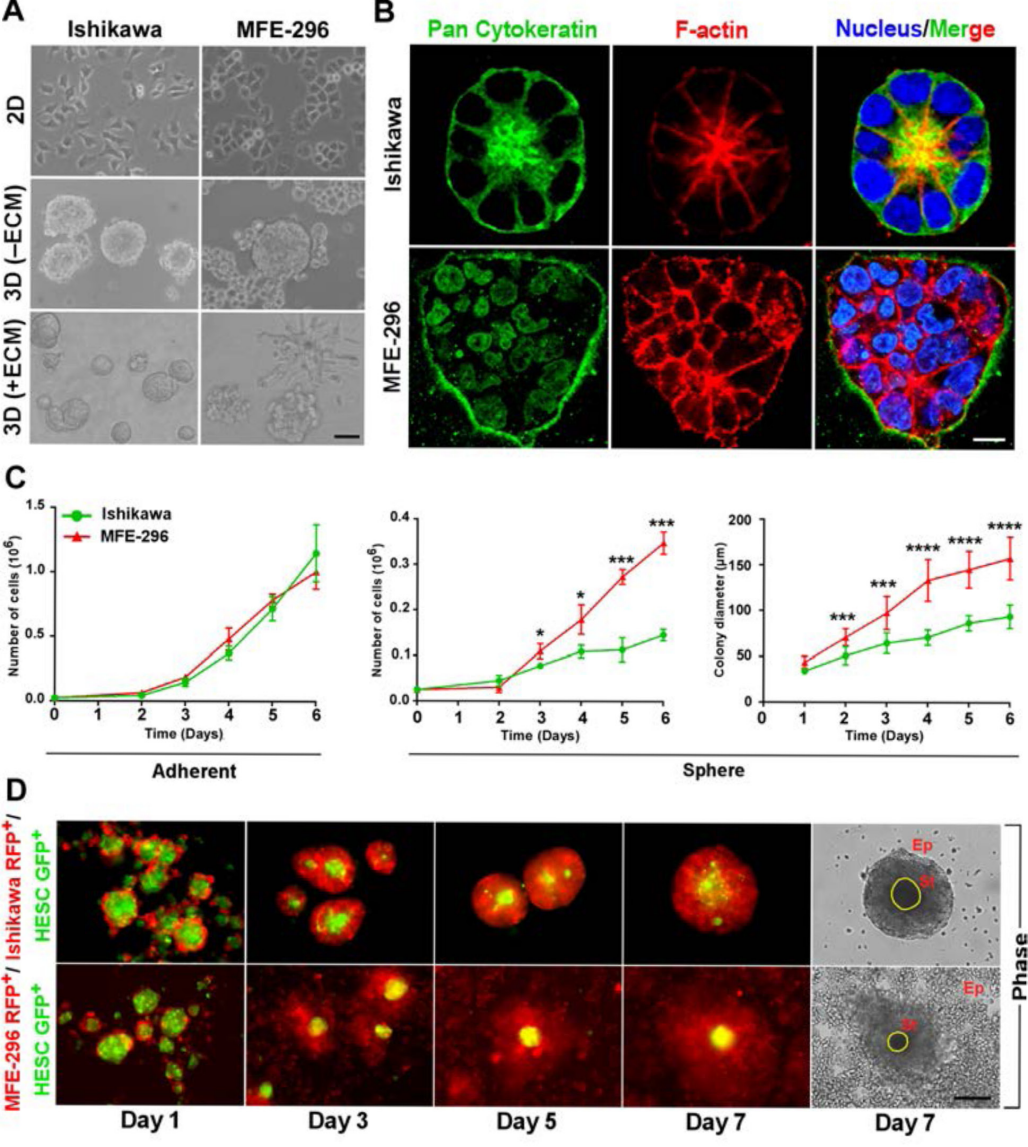
Endometrial cancer cells form distinct glandular and non-glandular pattern in reconstituted basement membrane and with endometrial stromal fibroblast co-culture **A.** Ishikawa and MFE-296 cells were grown as monolayers (top row), spheroids without ECM (middle row) and spheroids with ECM (bottom row), Phase contrast scale bar, 50 μm. **B.** Cells were cultured in RGF-BME for 7 days, stained for pan-cytokeratin (green), phalloidin (red), counterstained with Hoechst (blue), and imaged by confocal microscopy. One representative confocal section is shown out of 100 similar colonies. Phase contrast scale bar, 50 μm; confocal scale bar, 10 μm, Phase contrast scale bar, 50 μm. **C.** Comparison of cell proliferation and colony diameter between adherent (left) and spheroid (right) culture (*n* = 3). **D.** Fluorescence and phase contrast images of endometrial epithelial and fibroblast co-culture. Ep: Epithelial cells, St: Stromal cells. Scale bar, 200 μm. Error bars represent mean ± SD; ns = *P* > 0.05, *P < 0.05, **P < 0.01, ***P < 0.001, ****P < 0.0001.

To examine the contribution of microenvironment towards tumour heterogeneity, we cultured human endometrial cancer (EC) cell lines, Ishikawa and MFE-296 on plastic substratum (2D) and as 3D spheroids in absence and presence of reconstituted basement membrane matrix or ECM (Figure 1A). In contrast to monolayer culture, where every cell lines adopted loosely non-distinct morphologies, marked differences were acquired when grown on 3D ECM (Figure 1A). In 3D spheroid culture, Ishikawa forms glandular colony whereas MFE-296 forms non-glandular pattern of colonies (Figure 1A). To further investigate the cellular organization of each colony, we analysed confocal z-stack sections of individual colonies. Ishikawa 3D colonies have epithelial morphology (marked by pan-cytokeratin staining) and forms a central hollow lumen on day 7 of culture (marked by F-actin staining and nuclei organization) compared to MFE-296 non-glandular colonies (Figure 1B). Besides, Ishikawa and MFE-296 colonies have similar pattern of growth in 2D culture, but significant differences emerged in proliferation rate (2.4 ± 0.1 fold) and colony size (1.7 ± 0.4 fold) when grown on 3D matrix on day 6 (Figure 1C). This suggested that in 3D, under the influence of ECM derived cues, cells forming glandular structure undergo controlled growth and organize into polarized manner, whereas, cells forming non-glandular morphology proliferate more rapidly to develop disorganized aggregates.

To understand how endometrial stromal cells, a cell type primarily responsible for ECM deposition in the uterus [2, 21], influence the growth of endometrial epithelial cells, we developed a unique method of co-culturing these two cell types. We labelled endometrial epithelial cells and stromal fibroblast cells with RFP and GFP, respectively. Co-culture of epithelial and stromal cells revealed, non-glandular colony forming cells (MFE-296 RFP^+^) grow robustly around the stroma (HESC GFP^+^) but glandular colony forming cells (Ishikawa RFP^+^) have restricted growth with round morphology (Figure 1D). These results using different culture models provided evidence for substantial contributions of microenvironment or ECM towards cellular phenotypic diversity.

### TGF-β signalling pathway is upregulated in 3D non-glandular colonies

**Figure 2:**
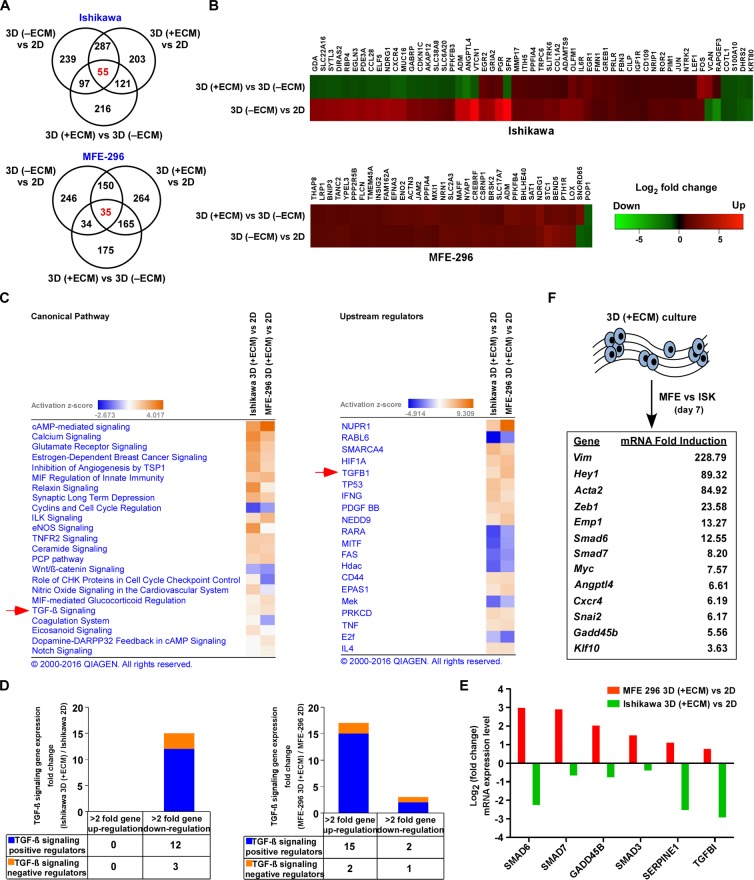
Gene networks and canonical pathways in EC monolayers vs 3D colonies **A.** Venn diagram representing genes differentially expressed (> 2-fold, *P* < 0.05) between varying culture conditions in Ishikawa (top) and MFE-296 (bottom) cells. **B.** Heat map showing hierarchical clustering of common differential expression profiles for Ishikawa (55 genes, top) and MFE-296 (35 genes, bottom) cells grown as 3D (−ECM) to monolayer or 3D (+ECM) to 3D (−ECM) culture (*P* < 0.05). Rows represent culture groups and columns represent individual genes. Each cell corresponds to the level of expression of a particular gene. Red represents an up-regulation and green a down-regulation in gene expression. **C.** Network of differentially expressed genes generated using the IPA bioinformatics tool was compared in 2D and 3D for canonical pathway (left) and upstream regulator (right) analysis and ranked by activation z-score. **D.** Stacked diagram represents number of TGF-β signalling genes upregulated (> 2-fold) or downregulated (> 2-fold) in Ishikawa (left) and MFE-296 (right) colonies in contrast to monolayer. **E.** Validation of gene expression profiles in 3D colonies vs monolayer of Ishikawa and MFE-296 cells by human TGF-β signalling pathway RT^2^ Profiler PCR Array. **F.** List of most strongly induced TGF-β signalling target genes after 7 days of 3D (+ECM) culture in MFE-296 (MFE) vs Ishikawa (ISK) cells.

To gain mechanistic insights into how endometrial cells respond to change in the microenvironment, we performed next generation RNA-Seq analysis on monolayer and 3D cultured spheroids. On day 7, cells completely form 3D structures with the unique cellular organization; therefore, we postulated this could be a critical time-point to see gene expression changes as compared to monolayer culture. RNA-Seq analysis identified 666 and 614 transcripts differentially expressed in Ishikawa (401 upregulated, 265 downregulated) and MFE-296 (426 upregulated, 188 downregulated) spheroids compared to 2D culture (> 2-fold change, *P* < 0.05) respectively (Figure 2A). In addition, we detected 55 and 35 most common genes in glandular (Ishikawa) and non-glandular (MFE-296) colony forming cells from adherent to spheroid culture and defined as ECM signature genes (Figure 2B). Ingenuity pathway analysis (IPA) of these genes revealed up and down regulation of several canonical cancer signalling pathways, such as estrogen-dependent breast cancer signalling, inhibition of angiogenesis by TSP1, Wnt/β-catenin, TGF-β and notch signalling and upstream target genes, such as NUPR1, RABL6, SMARCA4, HIF1A and TGFB1 in 3D glandular and non-glandular colonies (Figure 2C). However, both pathway and upstream regulator analysis displayed a comparable response of TGF-β signalling pathway in glandular and non-glandular 3D structures (Figure 2C). Out of the several pathways identified, TGF-β pathway has been known to be a major pathway in cancer progression and metastasis. This prompted us to further investigate whether ECM attached glandular and non-glandular colonies are regulated by TGF-β pathway with distinct metastatic propensity.

Notably, both TGF-β signalling pathway and upstream regulator TGF-β1 are more upregulated in non-glandular colonies (MFE-296; activation z-score, 4.008; *p*-value, 6.78E-06) compared to glandular colonies (Ishikawa; activation z-score, 2.188; *p*-value, 1.59E-12) (Figure 2C). Specifically, in accordance with the information contained in the IPA Knowledge Base (Ingenuity Systems), 17 TGF-β signalling related transcripts were upregulated (> 2-fold) in MFE-296 colonies vs monolayers, with 15 genes being known positive regulator of the pathway. However, 15 TGF-β signalling related transcripts (12 positive regulators) were downregulated (> 2-fold) in Ishikawa colonies vs monolayers (Figure 2D, Supplementary Table S1 and Supplementary Table S2). Notably, *Angptl4*, *Smad7*, *Twist1*, *Jun*, *Snai2* and *Vegfa* transcripts were upregulated (> 4-fold) in MFE-296 but not in Ishikawa, supporting an enrichment of TGF-β signalling target genes in 3D non-glandular colonies. To further verify the results from RNA-Seq data, quantitative RT-PCRs for the human TGF-β signalling pathway were performed and an overall upregulation of this pathway was detected in 3D colonies compared to monolayer culture (Supplementary Table S3 and Supplementary Table S4). As expected, TGF-β activated Smad-mediated transcriptional regulatory genes *Smad6*, *Smad7*, *Gadd45b*, *Smad3* and *Serpine1* were upregulated (> 2-fold) in non-glandular colonies whereas *Smad6*, *Serpine1* and *Tgfbi* genes were downregulated (> 2-fold) in glandular colonies confirming IPA analysis (Figure 2E). Notably, universal TGF-β responsive genes *Smad6*, *Smad7* and *Serpine1* have opposite expression pattern in 3D glandular and non-glandular colonies. An additional exploratory analysis of comparison of TGF-β signalling target genes between 3D (+ECM) cultured colonies of MFE-296 and Ishikawa demonstrated a hyper-activation of downstream genes in non-glandular vs glandular colonies (Figure 2F). Such observations of the pattern of gene expression correlate with dynamic phenotypic alteration of cells from adherent to spherical architecture.

### ECM proteins enhance TGF-β signalling in non-glandular colonies

**Figure 3:**
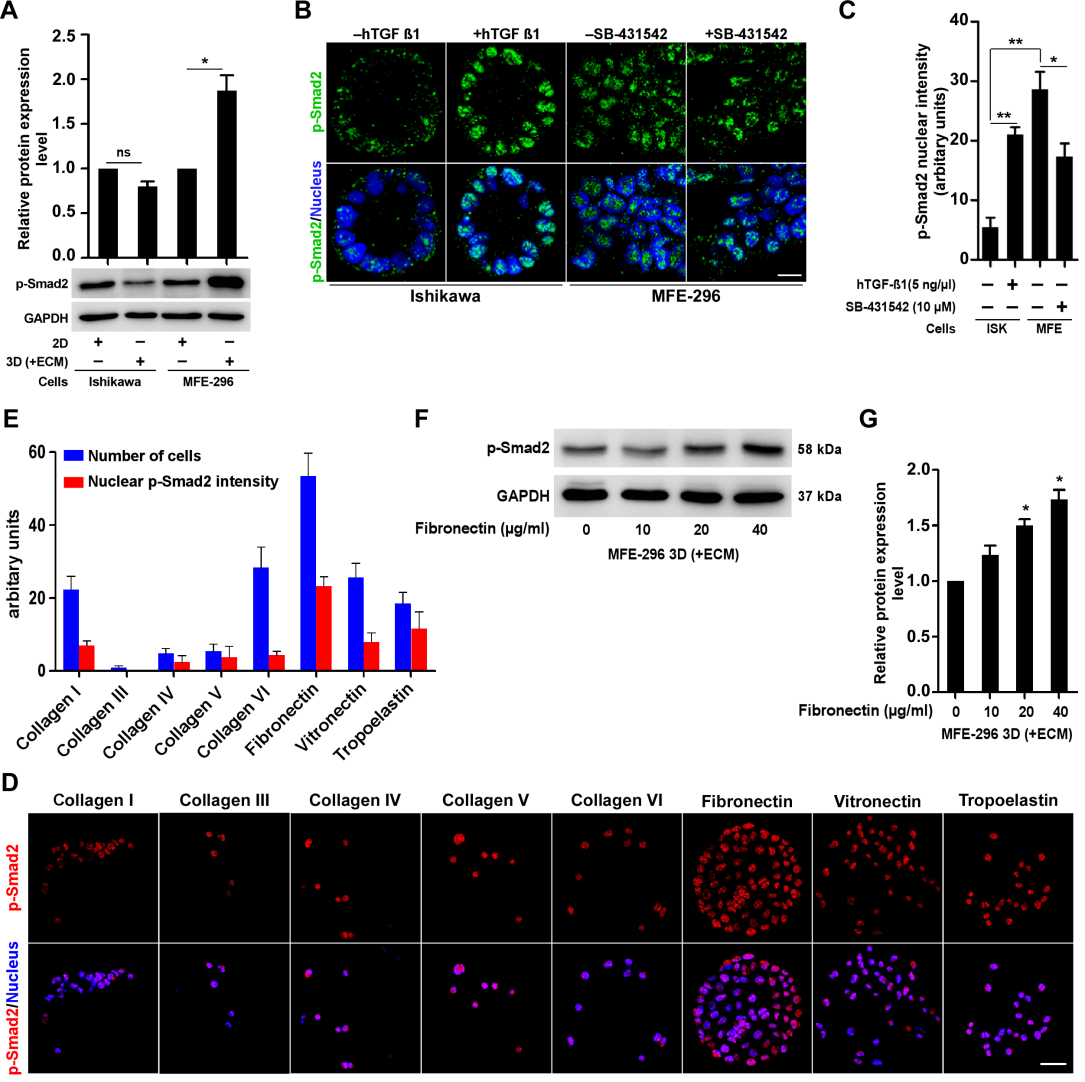
ECM protein, fibronectin modulates upregulation of TGF-β signalling via classical SMAD pathway **A.** Immunoblot for p-Smad2 protein, in 2D and 3D culture of Ishikawa and MFE-296 cells. Error bars are mean ± SD, n = 3; *P < 0.05. **B.** Confocal immunofluorescence analysis of p-Smad2 protein (green) in Ishikawa and MFE-296 cells after 72 hr post hTGF-β1/SB-431542 treatment. Nucleus stained with Hoechst (blue). Scale bar, 10 μm. **C.** Quantification of nuclear p-Smad2 fluorescence signal intensity in cells treated with hTGF-β1/SB-431542. Error bars are mean ± SD, *n* = 3; *P < 0.05, **P < 0.01. **D.** MFE-296 cells were cultured on MicroMatrix ECM array slide for 48 hr, stained for p-Smad2 (red) and counterstained with Hoechst (blue). Repeated twice with each having nine biological replicates. Scale bar, 50 μm. **E.** Quantification of nuclear p-Smad2 fluorescence intensity and number of cells grown on different ECM protein components. Data are shown as mean ± SD, *n* = 2. **F.** and **G.** Western blot and quantification for p-Smad2 in MFE-296 cells with increasing concentration of human fibroblast derived fibronectin are shown. Error bars represent mean ± SD, *n* = 3; *P < 0.05.

To find out whether the differences in TGF-β signalling activity encountered by mRNA analysis are reproducible at translational level, we investigated phosphorylation of Smad proteins (downstream molecules of TGF-β signalling pathway) in different culture environment (Figure 3A). Active TGF-β signalling leads to phosphorylation of SMAD proteins (Smad 2 and 3) [22]. We reproducibly detected significant upregulation (1.9 ± 0.3-fold) of p-Smad2 protein expression in 3D (+ECM) cultured MFE-296 cells (non-glandular) compared to monolayers whereas modest difference (0.8 ± 0.1-fold) was observed in case of Ishikawa colonies (glandular) (Figure 3A). We further ascertained expression of p-Smad2 protein in most of the EC cells forming glandular and non-glandular colonies (Supplementary Figure S1A). Reciprocal changes in p-Smad2 expression were observed, which was decreased in case of glandular colonies (ECC-1, HEC-1-B, MFE-280 and KLE) and increased in case of non-glandular colonies (COLO 684 and HEC-1-A) (Figure S1A). However, very low or non-detectable p-Smad2 expression was seen in two endometrial cell lines (RL95-2 and AN3 CA) in both 2D and 3D culture conditions (Supplementary Figure S1A). As ECM contact alters p-Smad2 expression, we addressed whether further activating or blocking TGF-β signalling pathway could alter the adaptive response of the 3D colonies. We treated these colonies with human TGF-β1 cytokine to activate the pathway and inhibited with SB-431542, which blocks TGF-β receptor I phosphorylation followed by downstream SMAD phosphorylation [23, 24]. In the 3D context, hTGF-β1 treatment increased p-Smad2 expression (2.2 ± 0.4-fold) in MFE-296 cells compared to basal level and co-treatment with SB-431542 abolished the expression (Supplementary Figure S1B). Similarly, increasing doses of hTGF-β1 and SB-431542 increased (*P* < 0.001) or decreased (*P* < 0.05) p-Smad2 expression in Ishikawa and MFE-296 cells, respectively (Supplementary Figure S1C). We confirmed these results by immunofluorescence staining of p-Smad2 protein (Figure 3B). At basal level, MFE-296 colonies displayed higher nuclear p-Smad2 (*P* < 0.01) fluorescence (bright green punctate) signal compared to Ishikawa colonies (Figure 3B). Compared to the basal level, treatment of hTGF-β1 and SB-431542 either increased or decreased the phosphorylated Smad protein expression in Ishikawa and MFE-296 colonies (Figure 3B and 3C). Collectively, these results suggest that TGF-β signalling has opposite activity in EC cells forming glandular and non-glandular colonies in 3D matrix. Sequestration of latent TGF-β in the ECM, as well as the dynamic interaction between multiple ECM components and latent TGF-β complexes, is crucial for activation of the latent cytokines [25, 26]. To explore the potential roles of distinct ECM proteins in regulating TGF-β signalling in EC cells, we assessed the nuclear p-Smad2 intensity of MFE-296 cells to different ECM components and relatively detected higher p-Smad2 protein expression from fibronectin-coated substratum (Figure 3D and 3E). Additionally, fibronectin relatively increased basal Smad2 phosphorylation (1.5 ± 0.1-fold) with an optimal concentration of 20 μg/ml (Figure 3F and 3G). These data suggest that fibronectin specifically induces Smad2 activation in EC cells.

### TGF-β signalling regulates conversion of glandular and non-glandular epithelium

**Figure 4:**
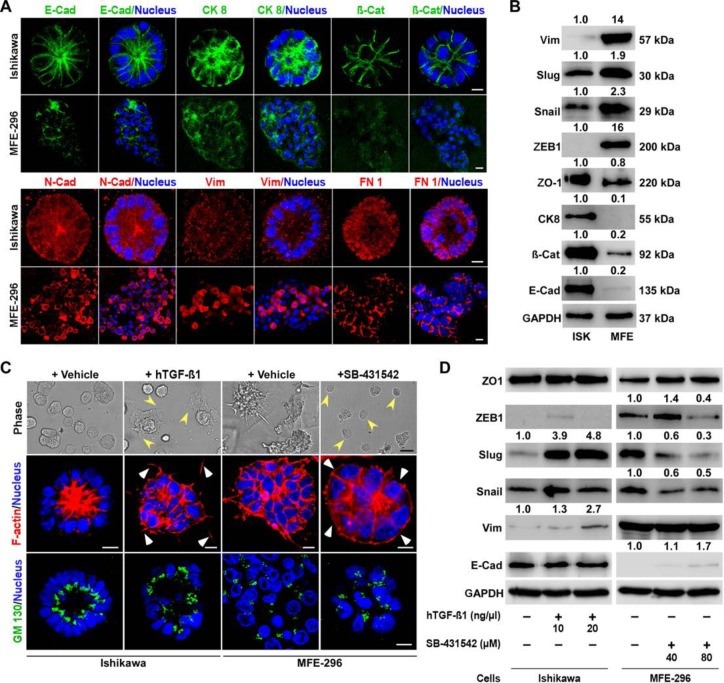
Differential expression of EMT markers in glandular and non-glandular endometrial cancer colonies **A.** Ishikawa and MFE-296 cells were cultured in 3D matrix, fixed on day 7, stained for E-Cadherin (green), Cytokeratin 8 (green), β-Catenin (green), N-Cadherin (red), Vimentin (red), Fibronectin 1 (FN 1) (red) and imaged by confocal microscopy. **B.** Several EMT markers were validated by western blot analysis of Ishikawa and MFE-296 3D colonies. **C.** Ishikawa and MFE-296 cells were cultured in 3D, serum starved and treated with hTGF-β1 or SB-431542 for 6 days. Cells were imaged by phase contrast, fixed, stained with phalloidin (red) for actin filaments and with GM130 (green) for polarity and imaged by confocal microscopy. **D.** Western blot of different EMT markers at indicated concentrations of hTGF-β1 and SB-431542 in Ishikawa and MFE-296 3D colonies. Nucleus stained by Hoechst (blue). Confocal scale bar equal, 10 μm; phase contrast scale bar, 200 μm.

TGF-β signalling is involved in many cellular functions, including EMT [27]. EMT in epithelial cells is a cellular plasticity process involving loss of cell-cell junction molecules and acquisition of spindle morphology with increased motility [28]. Based on our observations that TGF-β signalling is highly activated in non-glandular colonies, we postulated that there might be a discrete expression of EMT markers in colonies forming glandular and non-glandular morphology. Therefore, we analysed the expression of well-known markers of EMT in 3D colonies and detected epithelial markers (E-Cadherin, Cytokeratin 8 and β-Catenin) expressed at higher levels in Ishikawa colonies whereas mesenchymal markers (N-Cadherin, Vimentin and Fibronectin) expression were more prominent in MFE-296 colonies (Figure 4A). Furthermore, we tested expression of previously reported EMT markers [29], including Snail, Slug, ZEB1 and ZO-1, by western blot analysis in Ishikawa and MFE-296 (Figure 4B). As anticipated, this analysis showed that glandular colonies tend to express epithelial markers whereas non-glandular colonies with mesenchymal features were presented with a higher expression of mesenchymal markers.

To determine if TGF-β signalling activity is responsible for some of the mesenchymal features of non-glandular colonies, we tested the effect of TGF-β signalling agonist and antagonist on endometrial cancer colonies. Treatment of Ishikawa 3D colonies with hTGF-β1 stimulated actin disorganization with loss of polarity and acquiring of non-glandular features (Figure 4C). In contrast, inhibition of TGF-β signalling by treating MFE-296 colonies with SB-431542 restored the epithelial architecture (Figure 4C). Of note, the basic difference observed between glandular and non-glandular colonies was the polarity of the individual cells. To examine cell polarity in treated (hTGF-β1 or SB-431542) and untreated colonies, we performed immunostaining for GM130 [18] and found polarized (apical-basal) Ishikawa and non-polarized MFE-296 colonies reverted their phenotype after treatment (Figure 4C). We then verified whether conversion of glandular and non-glandular morphology by disruption of cell-cell adhesions was due to the altered expression of junctional proteins. No detectable changes were observed in the expression of ZO-1 protein either by hTGF-β1 or SB-431542 (Figure 4D). However, in Ishikawa colonies, vimentin expression relatively increased to 2.7-fold with hTGF-β1 treatment whereas in MFE-296 colonies, relative E-Cadherin expression increased to 1.7-fold and Snail, Zeb1 expression decreased to 0.5-fold and 0.4-fold with SB-431542 (Figure 4D). Interestingly, the expression of Slug protein was downregulated (0.3-fold) by SB-431542 in MFE-296 colonies, which was restored (4.8-fold) by hTGF-β1 in Ishikawa colonies in a dose-dependent manner (Figure 4D).

Collectively, the results show that TGF-β pathway activity is proportionately related to the expression of EMT markers and 3D structural organization of EC cells. The glandular architecture of endometrial cancer colonies is maintained by low TGF-β signalling. Slug seems to be a key protein which expression dramatically changes in EC cells that undergo either a TGF-β induced EMT or mesenchymal-epithelial transition (MET) by the inhibitor, suggesting that TGF-β pathway regulates morphological features of endometrial glands through slug EMT marker.

### Inhibition of TGF-β signalling impairs EC cell proliferation, invasion, matrix resistance and metastatic spread in non-glandular colony

**Figure 5:**
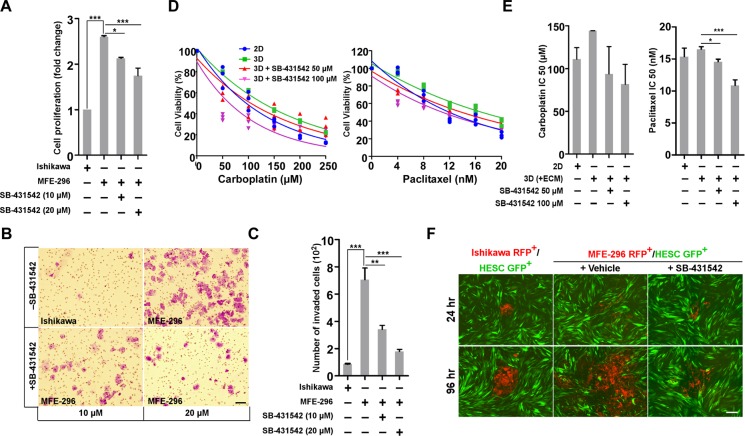
SB-431542 inhibits cell proliferation, invasion, chemo-resistance and metastasis *in vitro* **A.** Ishikawa and MFE-296 cells were cultured in 3D matrix, treated with SB-431542 at indicated concentration and assayed for cell proliferation (*n* = 3). **B.** Ishikawa and MFE-296 cells (1 x10^5^) were seeded onto the upper chamber of Transwell inserts and incubated for 24 hr with or without SB-431542 in the lower chamber to inhibit TGF-β signalling activity. Invasion of treated cells was determined by crystal violet staining. Cells in ten fields were imaged and counted to cover the entire filter in each group. **C.** Bar graphs represent the number of cells invaded in individual and treated groups (*n* = 3). **D.** Ishikawa and MFE-296 cells were cultured in monolayer and 3D (+ECM), treated with carboplatin (left) and paclitaxel (right) at indicated concentration with or without SB-431542 and were assayed for cell viability after 48 hr. **E.** IC50 values of carboplatin (left) and paclitaxel (right) were determined in different treatment groups from linear regression equation. **F.** Ishikawa RFP^+^ and MFE-296 RFP^+^ cells were grown in hanging drop 3D (+ECM) with or without SB-431542, spheroids transferred to monolayer of HESC GFP^+^ cells and time-lapse images were taken at indicated times. Scale bar, 200 μm. Error bars represent mean ± SD; *P < 0.05, **P < 0.01, ***P < 0.001.

The TGF-β signalling pathway is involved in a multitude of cellular processes, including cell proliferation, migration, and invasion [30]. Based on our observation that TGF-β signalling is activated in non-glandular colonies which also proliferate at a higher rate (2.6 ± 0.1 fold) than glandular one (Figure 5A), we hypothesized that inhibition of TGF-β signalling would suppress cell growth in 3D. Increasing concentration of SB-431542 significantly (*P* < 0.001) reduced proliferation of MFE-296 colonies compared to no treatment (Figure 5A). As expected, EC cells with more mesenchymal features (MFE-296) easily invade through the ECM barrier compared to cells with more epithelial characteristics (Ishikawa) (705 ± 12 MFE-296 cells vs 87 ± 5 Ishikawa cells; Figure 5B and 5C). SB-431542 treatment strongly attenuated (*P* < 0.001) invasion of MFE-296 cells in a dose-dependent manner (Figure 5C). At 10 μM concentration, only 163 ± 6 cells were able to invade to the lower chamber (Figure 5B and 5C). Recent work in many cancer types has shown that cancer cells in 3D microenvironment are less responsive to chemotherapeutic or targeted therapies [15, 31]. We also observed that EC cells are more sensitive to chemotherapeutic drug treatments on plastic substratum compared to 3D (+ECM) culture (Figure 5D). To address whether inhibition of TGF-β signalling would increase the efficacy of chemotherapeutic drugs against EC cells, we cultured MFE-296 cells in RGF-BME for 2 days followed by treatment with Carboplatin and Paclitaxel alone or in combination with SB-431542 for 72 hr (Figure 5D). The increased matrix resistance of MFE-296 cells was reduced in dual treatment with SB-431542 in a dose dependent manner, estimated from relative cellular viability and IC50 values (Figure 5D and 5E).

Hyper-activation of the TGF-β pathway is associated with induction of EMT and metastatic colonization of cancer cells in many organ systems [19, 32]. We have observed a higher level of TGF-β signalling activity and more mesenchymal characteristics in non-glandular colony forming EC cells compared to glandular colonies (Figures 3A and 4B), suggesting that these cells might have a higher metastatic potential. To test this, we stably transduced endometrial epithelial and stromal cells with lentiviral RFP and GFP, respectively. The RFP labelled epithelial cells were 3D cultured for 3 days to develop oncospheres and were transferred onto the layer of stromal cells (Figure 5F). Imaging was performed at regular intervals to determine the relative metastatic spread of individual colony (Figure 5F). As expected, MFE-296 cells spread at a higher rate compared to Ishikawa cells and treatment of oncospheres with SB-431542 significantly reduced the metastatic spread of MFE-296 cells in human endometrial stromal fibroblast cells, HESC (Figure 5F). In conclusion, these findings suggest EC invasion and metastasis is strongly regulated by TGF-β and inhibition of the signalling impairs tumour spread.

### Inhibition of TGF-β signalling delayed and decreased metastatic potential of EC cells *in vivo*

**Figure 6:**
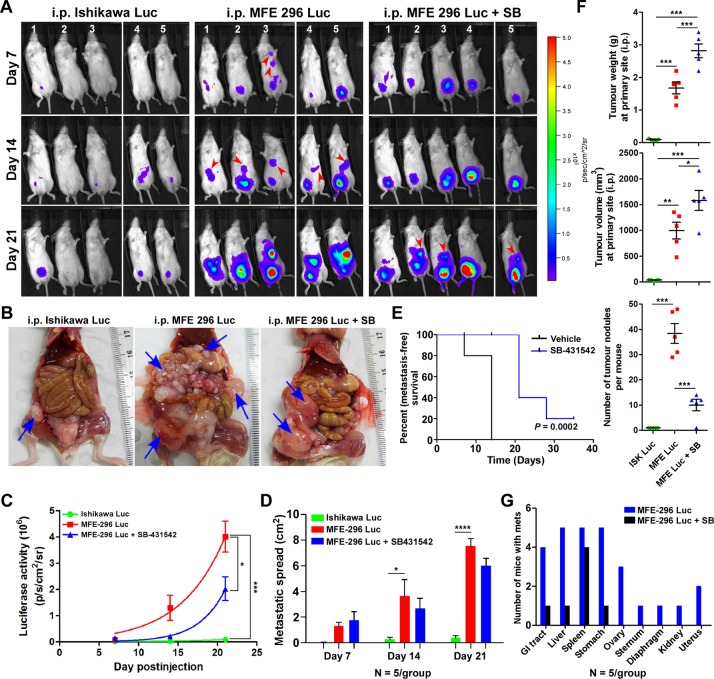
Inhibition of TGF-β signalling in an *in vivo* CDX model decreases metastatic spread **A.** Bioluminescent images of mice injected i.p. with luciferase tagged Ishikawa and MFE-296 cells with or without SB-431542 treatment, imaged at day 7, 14 and 21. BLI imaging conducted in total 5 mice/group/time point in two repeats (*n* = 3 and *n* = 2). **B.** Necropsy examination of xenograft mice for the evaluation of tumour burden and metastasis on day 35. **C.** Average radiance in each group at the indicated time points. **D.** Metastatic spread of tumour in each group of mice at the indicated time points. **E.** Shown is a Kaplan-Meier plot with or without SB-431542 treatment. **F.** *In vivo* growth of tumours after i.p. injection of Ishikawa and MFE-296 cells with or without SB-431542. Graphs depict tumour weight, volume and number of tumour nodules measured over 35 days. **G.** Number of mice bearing secondary tumours metastasized to different organs with or without SB-431542 treatment. Error bars represent mean ± SD, *n* = 5; *P < 0.05, **P < 0.01, ***P < 0.001, ****P < 0.0001.

Our *in vitro* results suggest that inhibition of TGF-β signalling during endometrial carcinogenesis may specifically reduce tumour invasion and metastasis *in vivo*. To test the effectiveness of inhibiting the TGF-β signalling pathway on *in vivo* tumour growth and metastasis*,* we developed EC cell-derived xenograft mouse models by intraperitoneal injection of Ishikawa^Luc^ or MFE-296^Luc^ cells in immunocompromised mice (Figure 6A). Both the cell lines were stably transduced with firefly luciferase and had equal luminescence emission (Supplementary Figure S2A and S2B). Compared to mice injected with Ishikawa^Luc^ cells, tumours in mice injected with MFE-296^Luc^ cells grew and metastasized at a higher rate (Figure 6A and 6B). Early metastasis was observed in mice bearing MFE-296^Luc^ tumours on day 7 (20%, 1/5 mice) and on day 14 (100%, 5/5 mice), whereas, no metastasis was observed in mice injected with Ishikawa^Luc^ cells even on day 21 (0/5 mice) (Figure 6A). Remarkably, SB-431542 treatment on mice injected with MFE-296^Luc^ cells caused marked inhibition of tumour cell metastasis up to day 14 and on day 21 late metastasis was observed (60%, 3/5 mice) (Figure 6A). Quantification of flux intensity values showed a decrease in luciferase activity and metastatic spread in mice treated with SB-431542 (Figure 6C and 6D). Furthermore, necropsy examination of the xenograft mice revealed only a solitary tumour attached to the peritoneum in the case of Ishikawa^Luc^ cells. However, metastatic spread and aggressive growth of tumours were present in mice injected with an equal number of MFE-296^Luc^ cells, which was inhibited by the treatment of SB-431542 (Figure 6B). Taken together, SB-431542 significantly improves metastasis free survival *in vivo* (Figure 6E).

Measurement of total tumour weight and volume revealed that mice treated with SB-431542 had an increased mean tumour weight (2.8 ± 0.4 g) and volume (1580 ± 385mm^3^) compared to the vehicle treated group mean tumour weight (1.7 ± 0.4) g and volume (997 ± 324) mm^3^ at the primary site (Figure 6F). However, a significant reduction in the number of metastatic cancerous growths was observed in SB-431542 treated mice (10 ± 4) compared to control mice (38 ± 8) (Figure 6F). Gross examination of mice injected with MFE-296^Luc^ cells revealed that these cells mainly metastasized to the liver, spleen, and stomach (100%) followed by GI tract (80%), ovary (60%), uterus (20%), kidney (10%) and distant metastasized to diaphragm (10%) and sternum (10%) (Figure 6G). SB-431542 treatment successfully reduced the metastasis of tumours, with tumours limited only to the GI tract (10%), liver (10%) and stomach (10%) (Figure 6G). Haematoxylin and eosin (H&E) staining of tumour samples confirmed that SB-431542 treatment decreased the metastasis of MFE-296 cells (Supplementary Figure S3A and Supplementary Figure S3B). Immunohistochemistry (IHC) of vimentin differentiated between metastasized tumour cells (with staining) from normal adjacent cells (without staining) (Supplementary Figure S3A). Collectively, these results showed that inhibition of TGF-β signalling suppresses EC metastasis, but not the primary tumour growth. However, treatment of mice with TGF-β inhibitor and chemotherapeutic drugs (SB-431542 and either carboplatin or paclitaxel) significantly reduced metastatic spread as well as primary tumour growth (Supplementary Figure S2C, S2D and S2E).

### Mouse EC xenografts and human EC patient samples showed similar response to TGF-β signalling during metastasis

**Figure 7:**
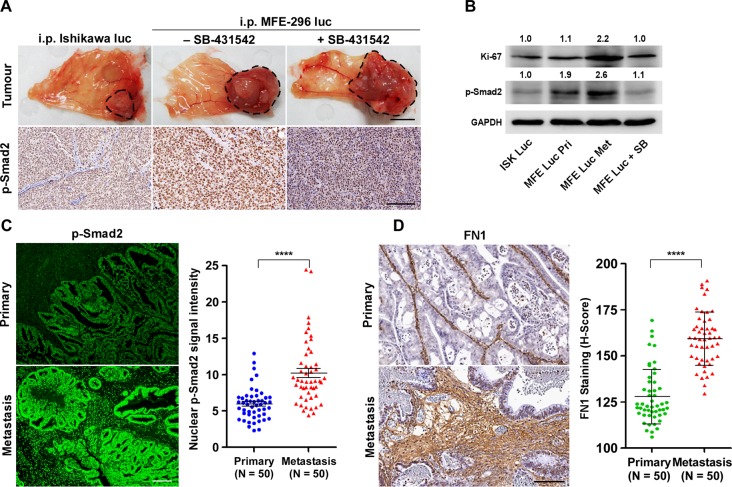
Up-regulation of TGF-β signalling at metastatic sites in an *in vivo* xenograft model and in fibronectin rich ECM of EC patient samples **A.** Primary necropsy tumours attached to peritoneum from xenograft mice without or with SB-431542 treatment were processed for p-Smad2 IHC (brown staining). Organ image scale bar, 1 cm; IHC scale bar, 100 μm. **B.** Western blot analysis of vehicle and SB-431542 treated primary (Pri) and metastatic (Met) tumour samples. Ishikawa (ISK) and MFE-296 (MFE). **C.** Tissue sections from primary (Uterine epithelium) and metastatic sites (different body organs) of EC patients were analysed for p-Smad2 immunofluorescence (green) and representative lesions are shown. Quantification of p-Smad2 protein expression is shown, *N* = 50 (Same patient or age matched), Mann-Whitney U test, ****P < 0.0001. **D.** Immunohistochemistry staining of fibronectin (FN1) on primary and metastatic human patient tissue samples. Scale bar, 100 μm. Quantification of FN1 staining intensities was shown as H-Score, N = 50 (Same patient or age matched), Mann-Whitney U test, ****P < 0.0001.

Next, we examined whether metastatic propensity of EC cells is proportionately related with TGF-β pathway activation. We isolated primary and distant metastatic tumour samples from xenograft mice bearing tumours of Ishikawa and MFE-296 cells with or without SB-431542 treatment (Figure 7A). Gross and H&E staining analysis of tumour samples revealed that in all groups, primary tumours developed on the peritoneal wall near to the injection site (Figure 7A), which might have later metastasized to different abdominal organs. IHC of p-Smad2 protein on full-face sections taken from the edges of tumours adjacent to peritoneum lining manifested high expression of p-Smad2 protein (Figure 7A). As compared to the vehicle treated mice, SB-431542 treatment decreases p-Smad2 expression level at the primary site (Figure 7A). Protein lysates from SB-431542 treated MFE-296 tumours showed an expected decrease in Smad phosphorylation, whereas higher p-Smad2 expression was observed in MFE-296 primary tumours (1.9-fold) and metastatic tumours (2.6-fold) compared to Ishikawa tumours at the primary site (Figure 7B). A similar correlation was observed with the expression of a cell proliferation marker, Ki-67 (Figure 7B). Interestingly, Ki67 expression analysis also demonstrated that metastatic tumour cells proliferate at a higher rate (2.2-fold) compared to primary tumours (Figure 7B).

**Figure 8:**
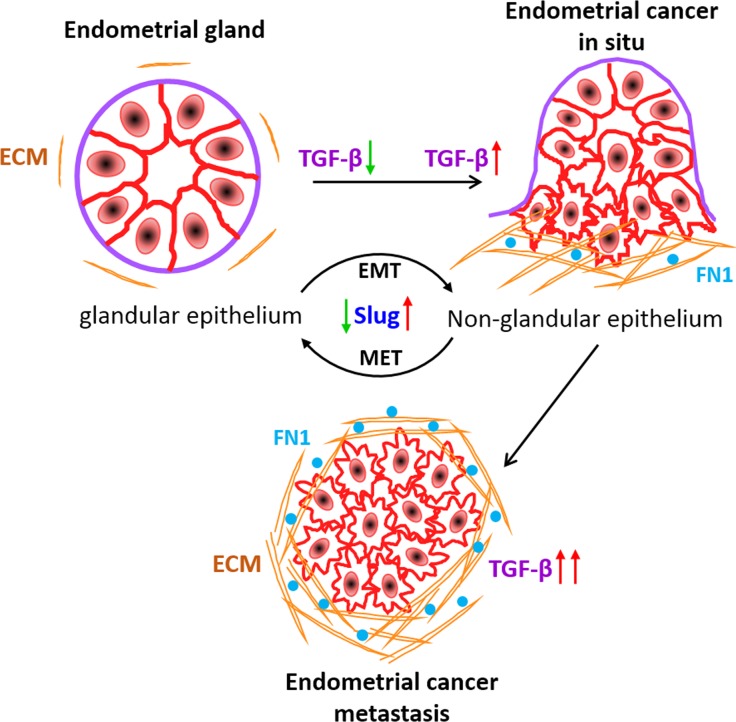
Working model of ECM derived TGF-β signalling during EC metastasis Active TGF-β signalling sustains malignant transformation of EC cells and metastasis. Slug expression increases with the oncogenic transformation of acinar architecture of cells to non-glandular invasive morphology. Fibronectin in ECM deposit of in situ carcinoma relatively facilitates TGF-β signalling to promote EC metastasis.

To address whether changes in TGF-β signalling that we have observed in EC cells and xenograft models also correlate with observations in human patients, we performed p-Smad2 protein expression analysis on human EC patient tissue samples (Figure 7C). Comparison of fluorescence p-Smad2 signal intensity between primary tumour (limited to the uterus) and metastatic cancer (other organs) from 50 (same patients, *N* = 33; age matched, *N* = 17) human patients revealed significant (*P* < 0.0001) increase in p-Smad2 protein expression at the metastatic sites (Figure 7C, Supplementary Table S5). Of note, we observed more deposition of fibronectin protein in human metastatic cancer patient samples (Figure 7D) concurring *in vitro* results (Figure 3F). These data support our hypothesis that upon loss of ECM integrity, the defective ECM with high fibronectin protein activates the latent TGF-β signalling to promote EC metastasis (Figure 8).

## DISCUSSION

Normal epithelial cells in the human uterus organize into an inner single layer of columnar epithelium as endometrium and into acinar structures in the stroma as endometrial glands [33]. Endometrial hyperplasia or cancer develops with the disruption of the acinar architecture of endometrial glands [34] and can be categorized as a hallmark of cancer. Epithelial cells of endometrium maintain close contact to each other through cell-cell junction proteins whereas invasive endometrial carcinoma is characterized by disorganized cells with aberrant or lack of polarization. Using a robust organotypic 3D culture model that approximates formation of human endometrial glands, we characterize normal and invasive carcinoma phenotypes. By profiling gene expression of endometrial cancer cells in the different environment, we have uncovered a major signalling pathway which is differentially expressed in glandular and non-glandular endometrial cancer colonies.

Our study began with a transcriptional dichotomy between two single cell heterogeneity in a relevant ECM context. Two-dimensionally cultured cells in contact with basement membrane matrix undergo a robust change in gene expression defined by TGF-β signalling pathway. Alteration of TGF-β pathway and downstream target genes in ECM attached cells probably occurs by post-transcriptional signalling from TGF-β family receptors. We speculate ECM proteins might bind to TGF-β ligands or receptors and exist as latent complexes or act as stimulators of the pathway [25], this requires further investigation. Moreover, we have found upregulation of TGF-β pathway in invasive endometrial cancer colonies growing on fibronectin substratum in a dose dependent manner. Given that fibronectin acts as one of the major ECM components which supports the metastatic niche [35], may explain the relative activation of TGF-β pathway on fibronectin surface to promote metastasis.

Proper dynamic regulation of the pathway is critical for establishing and stabilizing the identity of the acinar and invasive morphology of ECM attached endometrial cells. Supporting the beneficial function of TGF-β signalling inhibition in maintaining acinar organization of endometrial cancer colonies, activation of the pathway in EC cells disrupted cell polarity complex protein, GM130 and cytoskeletal organization protein, F-actin. In contrast, inhibition of the pathway in invasive colonies reversed the expression of polarity proteins. Interestingly, TGF-β pathway induced EMT proteins have contrasting levels of expression in both types of colonies. As reported earlier, EMT promotes invasiveness of cancer cells, suggesting together with our results that non-glandular colonies with mesenchymal features are more prone to metastasize. Moreover, out of several EMT proteins, we have found dynamic fold change of slug protein during activation and suppression of the TGF-β pathway in two groups of colonies respectively. Thus, slug might be a major targeted protein during the transition of differentiated to malignant endometrial cell behaviour.

Worldwide, endometrial cancer is the leading cause of cancer death in women, with most morbidity and mortality resulting from the metastatic disease [36, 37]. Additionally, the incidence of endometrial cancer continues to rise with increasing obesity rates worldwide and is likely to become one of the major obesity related cancer issues in the next 2 to 3 decades [38]. Currently, patients with metastatic endometrial cancer have a poor prognosis which might be due to chronic upregulation of TGF-β pathway. Our results on the histopathological analysis of human endometrial cancer patients show a strong correlation of active and upregulated TGF-β pathway at metastatic sites of cancer compared to primary origin of tumour in the uterus. These studies suggest that TGF-β enhances tumour progression and metastasis in endometrial cancer. In most metastatic cancers, cells first become resistant to TGF-β induced growth inhibition and later high levels of TGF-β can promote cancer progression in an autocrine and/or paracrine manner that favours invasion and metastasis [19, 39]. But how TGF-β pathway gets upregulated in malignant tumours has been enigmatic. Our data provides renewed emphasis on the ECM component of the microenvironment that regulates dynamic asynchronicity of TGF-β signalling in constituent EC cells. In the present study, we have demonstrated that SB-431542, a novel ALK5 receptor kinase inhibitor, inhibits tumour invasion and abrogated the pro-oncogenic functions of TGF-β including EMT and metastasis in both *in vitro* and *in vivo* models. Although further studies are required, our initial *in vivo* xenograft studies revealed, TGF-β inhibitor decreases the tumorigenicity of the highly aggressive MFE-296 cells in a murine EC model. Taken together, these results support the significant contribution of the TGF-β pathway in EC metastasis and suppression of the pathway might reduce the metastatic spread of cancer.

Developing a new therapeutic strategy by targeting TGF-β signalling might be promising to block TGF-β pathway in advanced stages of invasive and metastatic EC. In fact, the ECM dependent upregulation of TGF-β signalling pathway required for tumour cell growth is restricted to the specific niche of tumour population. By extension, blocking the signal from microenvironment of constituent tumour cells might be useful in suppressing tumour growth outside their normal niche.

## MATERIALS AND METHODS

### *In vivo* cell line derived xenograft (CDX) experiments

Six to eight weeks old female NOD/SCID/γ mice (Jackson lab), housed in pathogen free conventional cages on a 12 hr light-12 hr dark cycle, fed *ad libitum* were used for tumorigenicity assays. All procedures for mice experimentation were approved by the University of Newcastle Animal Care and Ethics Committee. Luciferase labelled Ishikawa and MFE-296 cells (1.5 × 10^6^) in 200 μL of 1:1 sterile DPBS and Matrigel were injected intraperitoneal (i.p.) into lower abdomen region of female NOD/SCID/γ mice (5/group). Mice were injected i.p. thrice per week with SB-431542 (100 μg/kg), carboplatin (15 mg/kg) and paclitaxel (5 mg/kg) alone or in combination. The endometrial cancer cells were genetically engineered to express the firefly luciferase gene to quantitatively track *in vivo* growth and metastatic potential. Tumour development and metastatic spread were assessed weekly by monitoring luciferase signal using an IVIS bioluminescent imaging system (PerkinElmer) with a standard protocol over a period of 21 days. Animal health was monitored by daily observation and weekly assessment of weight. After 4 weeks, mice were euthanized and metastatic spread of cancer cells was assessed by counting the number of tumour nodules within the peritoneal cavity of each mouse. Recovered tumours from primary and metastasized sites were fixed in formalin, embedded in paraffin and sections stained with haematoxylin and eosin (H&E) and against antibodies to analyse tumour pathology.

### Primary and metastatic patient samples

Human endometrial cancer patient samples (Primary and Metastatic) were collected at the John Hunter Hospital, Newcastle, NSW, Australia and obtained through the Hunter Cancer Tissue Biobank, University of Newcastle. The protocol was approved by the Institutional Human Research Ethics Committee of the University of Newcastle. Consent from patients was obtained as per the approved guidelines. Tumour from uterus was collected as primary and from different body parts wherever cancer has spread as the metastatic sample. In case of unavailable metastatic tissue, age matched metastatic samples were collected for this study. Detailed patient's information was listed in Supplementary Table S5.

### 3D glandular morphogenesis assay

Endometrial cancer cells were cultured on top of a thin layer of reduced growth factor basement membrane extract (Cultrex^®^ RGF BME: Trevigen) purified from Engelbreth-Holm-Swarm (EHS) tumour, as previously described with minor modifications [40]. Briefly, BME was plated onto each well of a Falcon eight-well culture slide (*In Vitro* Technologies), allowed to solidify for 20 minutes at 37°C incubator, and subsequently overlaid with 400 μl of complete medium containing 1 × 10^4^ trypsinized cells and 3% RGF BME. Cells were cultured for 7 days with media change every 2 or 3 days and harvested for protein and RNA isolation or imaging of 3D morphology by confocal microscopy (Olympus FluoView FV1000). Cells were also grown in three dimensions in absence of RGF BME on poly-HEMA coated (15 mg/ml in 95% EtOH and allowed to dry completely) 24-well plates for growth curve comparison.

### RNA-Seq and data analysis

Total RNA was isolated from 2D monolayer and 3D grown spheroids using RNeasy Mini kit (Qiagen) following manufacturer's instructions. Library preparation was performed from total RNA using the TruSeq Stranded total RNA sample preparation kit (Illumina). Sequencing was carried out by the Australian Genome Research Facility on an Illumina HiSeq using HT version 4 chemistry with 50 bp single-end reads. A minimum of 25 million reads was achieved per sample. Raw FASTQ files were analysed using FASTQC (version 0.10.1) and adaptor contaminations were removed using Cutadapt (version 1.12). Raw reads were mapped using TopHat2 (version 2.0.13) to reference genome hg19. Mapped reads were subjected to cufflinks (version 2.2.1) for transcript assembly. Differential gene expression was determined using cuffdiff.

### Cell culture and lentiviral transduction

Human endometrial cancer cell lines were cultured in MEM (HyClone) supplemented with 10% heat-inactivated fetal bovine serum (FBS, Bovogen Biologicals). Human endometrial stromal fibroblast cell line T HESCs (ATCC^®^ #CRL-4003^TM^) was maintained in DMEM:F12 (Sigma) without phenol red, supplemented with 10% charcoal-stripped FBS (Invitrogen). The cells were propagated in tissue culture flasks in respective growth medium containing 2mM L-glutamine (HyClone) and antibiotics (50 units/mL penicillin, 50 mg/l streptomycin; Gibco) at 37°C and 5% CO_2_. STR profiling was performed for cell authentication and all the cell lines were tested mycoplasma negative.

Firefly Luciferase (GeneTarget Inc #LVP434), RFP (GeneTarget Inc #LVP428), and GFP (Qiagen #CLS-PCG) expressing stable (pooled antibiotic-resistant population) endometrial epithelial and stromal cell lines were established by transduction of lentiviral particles according to manufacturer's instructions.

### *In vitro* metastatic spread assay and time-lapse imaging

RFP-expressing endometrial cancer cell spheroids were grown by culturing 100 epithelial cells (Ishikawa RFP^+^ or MFE-296 RFP^+^) in hanging drop fashion [41] with 3% matrigel. After 72 hr, oncospheres were transferred to 90% confluent monolayer of GFP-expressing endometrial stromal fibroblast cells (HESC GFP^+^). Images were obtained every 24 hr using 10x objective on JuLi^TM^ Stage Real-Time Cell History Recorder (NanoEnTek) in an incubator at 37°C and humidified 5% CO_2_.

### 3D immunofluorescence staining and microscopy

Extracellular matrix (ECM) embedded 3D colonies were fixed in 4% paraformaldehyde (PFA, Electron Microscopy Sciences, ProSciTech) for 20 minutes and processed for immunofluorescence, as previously described with minor modifications [40]. Briefly, colonies were permeabilized with 0.5% Triton X-100 in PBS for 10 minutes at 4°C, blocked in immunofluorescence buffer (130 mM NaCl, 7 mM Na_2_HPO_4_, 3.5 mM NaH_2_PO_4_, 0.1% BSA, 0.2% Triton X-100, 0.05% Tween 20) containing 10% goat serum and incubated overnight at 4°C with the indicated dilutions of primary antibodies. Next day, cells were washed thrice with TBS, 0.1% Tween 20, 10 minutes each and incubated with 0.5% Triton X-100 in TBS containing 5 μg/ml of Hoechst 33342 (Invitrogen) and 1:250 dilution of Alexa Fluor secondary antibodies (Jackson ImmunoResearch Laboratories) for 1 hr at room temperature. Immunofluorescence staining was visualized on a confocal LASER scanning microscope (FV1000, Olympus) using the oil-immersion 40x magnification objective and analysed with Fluoview FV10-ASW 1.7 software. 3D images were shown as mid-structure from z-stack sections.

### Cellular proliferation assay

For comparison of cellular proliferation and viability, 5000 cells were seeded in 100 μL complete medium per well of 96-multiwell flat bottom plates (Corning Costar) for 2D and on basement membrane ECM-coated plates for 3D and incubated for 24 hr at 37°C, 5 % CO2 for cells to adhere. Cells were then treated with indicated concentrations of carboplatin, paclitaxel and SB-431542 followed by further incubation of 72 hr. At the end of each incubation period, cell viability was examined by CellTiter-Glo^®^ Luminescent cell viability assay (Promega) according to the manufacture's protocol and IC50 values were calculated.

### Transwell cell invasion assay

For invasion assay, each well of Transwell assay inserts (6.5 mm diameter, 8 μm pores, Sigma #CLS3422) was coated with 0.5 mg/ml (1:30 dilution) of Matrigel (Cultrex^®^ RGF BME: Trevigen) with ice cold sterile DPBS, and 100 μL of this slurry was pipetted into each insert of the invasion assay plate. The plate was incubated at 37°C for 2 hr and then dried at room temperature (25°C) overnight under sterile conditions. Ishikawa and MFE-296 cells (1 × 10^5^) were serum starved for 16 hr, resuspended in MEM medium and placed in the upper chamber, whereas 10% FBS-MEM alone or containing SB-431542 with the indicated concentration was added to the lower chamber. After 24 hr incubation at 37°C invaded cells were fixed in 70% alcohol and stained with crystal violet (0.5%) for 10 minutes. Matrigel and non-invading cells were mechanically wiped using cotton swabs. Cells were imaged and quantified using ImageJ software.

### Western blot analysis

Cells cultured in 3D and 2D were harvested and lysed in ice-cold RIPA (radio immunoprecipitation assay) buffer (50 mM Tris-HCl pH 7.5, 150 mM NaCl, 1% NP-40, 0.5% Sodium deoxycholate, 0.1% SDS) containing protease and phosphatase inhibitors (Sigma). Lysates were purified by centrifugation at 12,000 rpm for 10 minutes at 4°C and supernatant was collected. Purified lysates were boiled in 1x Laemmli sample buffer (0.04 M Tris-HCl pH 6.8, 0.2% SDS, 0.01% bromophenol blue, 10% β-mercaptoethanol and 10% glycerol) for 5 minutes at 95°C. Aliquots of cell lysates containing equal protein mass were resolved by 10% SDS-PAGE gels, transferred to nitrocellulose blotting membranes (GE Healthcare Life Sciences), blocked with 5% skim milk (w/v) in TBS (20 mM Tris, 150 mM NaCl, pH 7.5), 0.1% Tween 20, for 1 hr at room temperature and probed with primary antibodies at the recommended dilutions for overnight incubation at 4°C. Subsequently, membranes were probed with relevant secondary antibodies conjugated with horseradish peroxidase (Jackson ImmunoResearch Laboratories) for 1 hr at room temperature. After washing, western blot membranes were developed using chemiluminescent substrate for detection of HRP (Millipore) and proteins were detected by chemiluminescence (Fujifilm LAS-4000). Quantification of the mean pixel density of the protein bands was determined using NIH ImageJ plugin.

### Human TGF-β signalling array

Total RNA was isolated from Ishikawa and MFE 296 cells grown as monolayer and 3D colonies, using RNeasy Mini kit (Qiagen) following manufacturer's instructions. 500 ng of total RNA was used for the cDNA synthesis using RT^2^ First Strand Kit (Qiagen). Quantitative real-time PCR (Q-PCR) was performed using RT^2^ SYBR Green ROX qPCR Mastermix and RT^2^ Profiler PCR Array kit (Qiagen) on 7900 HT FAST Thermocycler (Applied Biosystems). Amplification and analysis were performed as per manufacturer's instructions. Relative quantification [comparative Ct (ΔΔCt) method] was used to compare the expression level of the test genes with the internal control (arithmetic mean of five housekeeping genes included in the array). Fold change expression of genes in 3D (+ECM) and 2D culture were plotted in log scale range.

### IHC and IF

Mouse EC xenograft tumours were fixed with 4% paraformaldehyde overnight, followed by embedding and sectioning. Human EC patient-derived primary and metastatic tumour sections were deparaffinised in xylene followed by rehydration. Antigen retrieval was performed using sodium citrate buffer (10 mM Tri-sodium citrate, 0.05% Tween 20, pH 6.0). Slides were washed with TBS with 0.1% Tween 20 and incubated with 0.3% H_2_O_2_ to block endogenous peroxidase. Sections were blocked with 10% goat serum with 1% BSA in TBS containing 0.1% Triton X-100 for 1 hr at room temperature. After blocking, the sections were incubated with primary antibodies, followed by peroxidase-conjugated secondary antibodies (Thermo Fisher Scientific) and DAB substrate (Sigma) to detect bound antibodies. For quantification, slides were digitized at 20x absolute resolution using an Aperio AT2 scanner. Quantitative IHC analysis was performed using the Halo^TM^ image analysis platform and the pixel intensities of DAB staining were calculated using the Area Quantification v1.0 algorithm (Indica Labs, New Mexico, USA). Immunohistochemistry intensity score (H-Score) was calculated as described previously [42]. For IF, tissue sections were incubated with primary antibodies, followed by Alexa Fluor secondary antibodies (1:250; Jackson ImmunoResearch Laboratories) to detect fluorescence signal. Images were taken at 10x magnification objective with 400 ms exposure time on a fluorescence microscope (Olympus DP72) using cellSens Standard software. Quantification of nuclear fluorescence signal was performed using the “Intensity Ratio Nuclei Cytoplasm Tool” plugin of Image J (NIH, USA).

### Statistics

Statistical analyses were performed using GraphPad Prism 6.0 software. The results were presented as mean ± SD. Statistical significance was determined using a two-way analysis of variance and Bonferroni post-test unless otherwise indicated. A *P* value of < 0.05 was considered statistically significant.

## SUPPLEMENTARY MATERIALS FIGURES


