# Supplementary material for: Inhibition of extracellular matrix mediated TGF-β signalling suppresses endometrial cancer metastasis
Source: Oncotarget. 2017 May 22;8(42):71400–17. doi: 10.18632/oncotarget.18069 (PMC5641058; doi:10.18632/oncotarget.18069)
Supplement: Supplementary file 1 [file oncotarget-08-71400-s001.pdf]

Inhibition of extracellular matrix mediated TGF-β signalling suppresses endometrial cancer metastasis

Supplementary Material

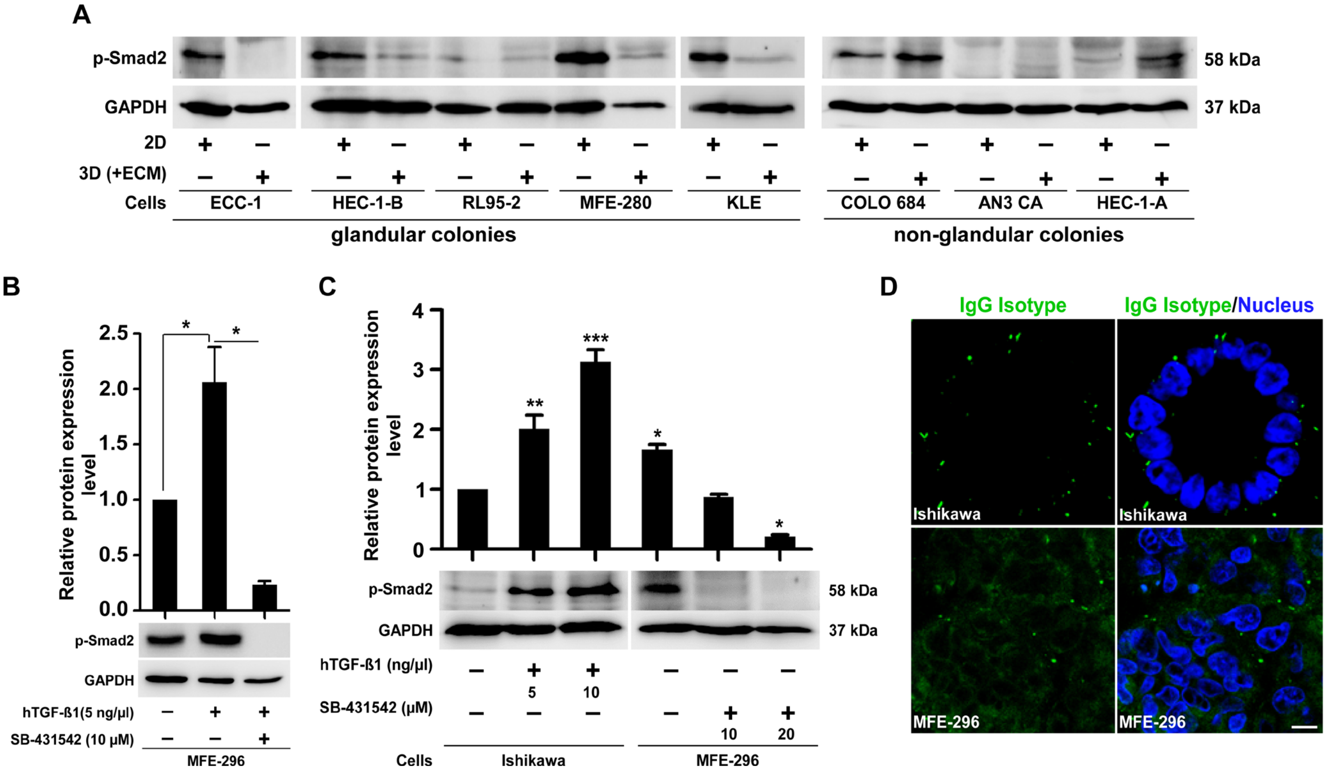

Supplementary Figure S1: Up-regulation of TGF-β signalling pathway in non-glandular endometrium colonies (A) Western blot of p-Smad2 protein in 2D and 3D matrix culture of endometrial cancer cells forming glandular and non-glandular colonies**.** (B) Immunoblot for p-Smad2 protein in MFE-296 cells after 72 hr post hTGF-β1/SB-431542 treatment in 3D (n = 3). (C) Immunoblot for p-Smad2 in Ishikawa and MFE-296 cells with increasing doses of 72 hr hTGF-β1/SB-431542 treatment (n = 3). (D) Confocal immunofluorescence analysis of Ishikawa and MFE-296 cells using concentration matched Rabbit mAb IgG Isotype control (green), DNA (blue). Scale bar, 10 µm. Data are shown as mean ± SD; ns = $P > 0.05$, *$P < 0.05$, **$P < 0.01$, ***$P < 0.001$.

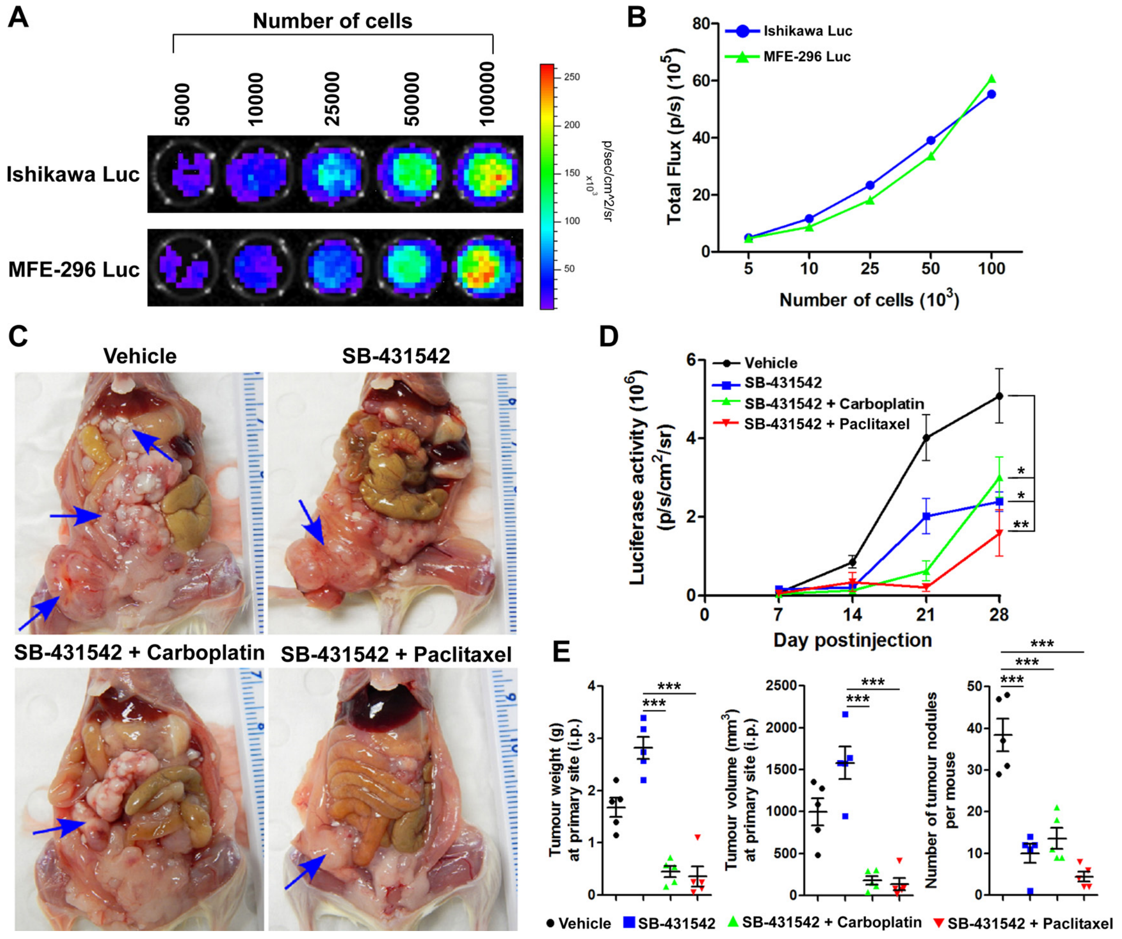

Supplementary Figure S2: In vitro luciferase activity of EC cells and inhibition of tumour growth and metastasis in vivo

(A) Bioluminescent imaging of Ishikawa^{Luc} and MFE-296^{Luc} cells after stable transduction of firefly luciferase. (B) Graph showing an estimation of total flux intensity in both the cell lines with increasing cell number. (C) Necropsy examination of xenograft mice (injected i.p. MFE-296^{Luc} cells) with dual treatment (SB-431542 and either carboplatin or paclitaxel), for evaluation of tumour burden and metastasis on day 35. (D) Average luciferase activity in each group at indicated time points. (mean ± SD, n = 5; *$P < 0.05$, **$P < 0.01$) (E) Graphs depict tumour weight, volume and number of tumour nodules measured over 35 days at indicated groups. Error bars represent mean ± SD, n = 5; ***$P < 0.001$.

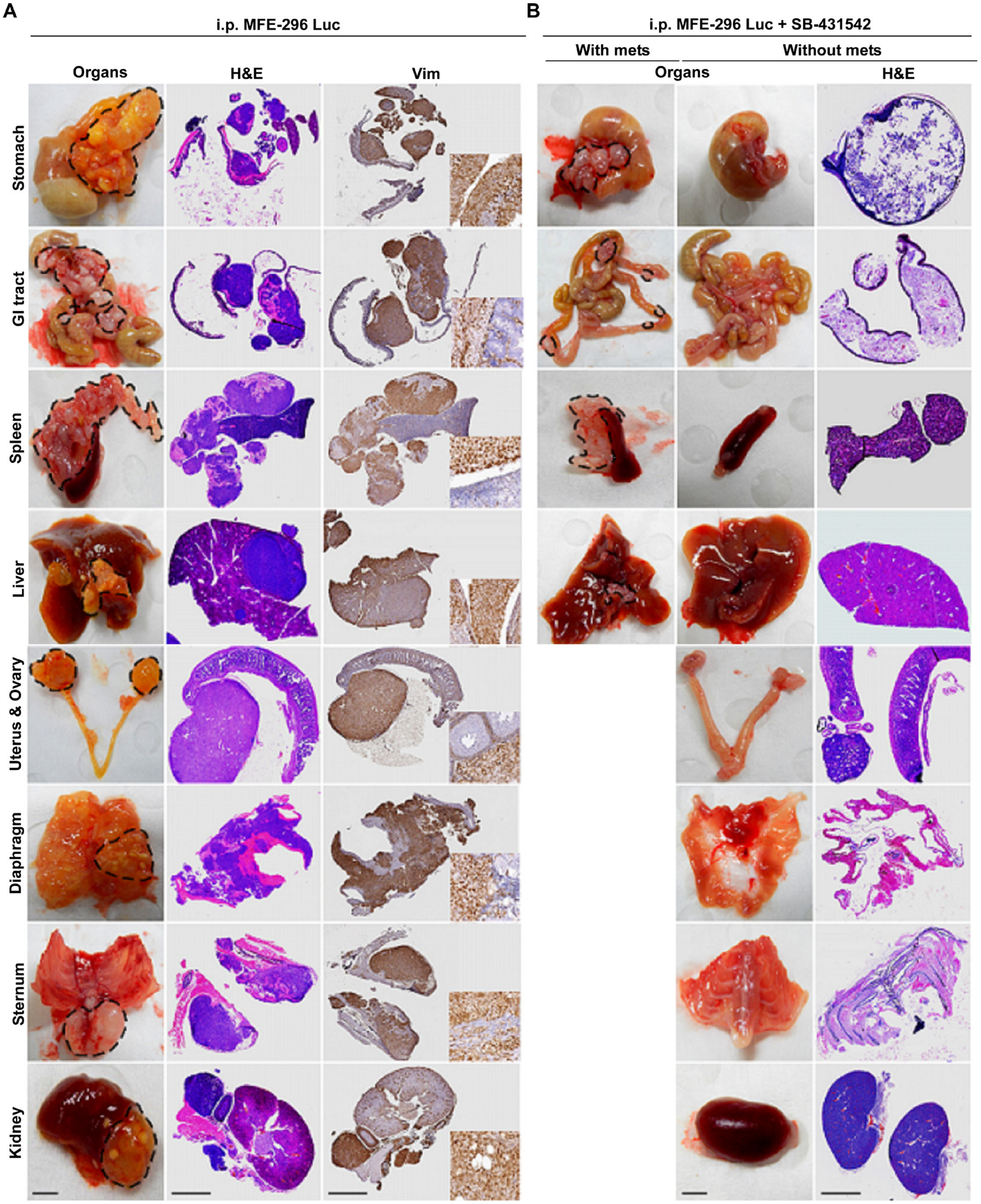

Supplementary Figure S3: NOD/SCID/γ mice bearing heterotopic xenograft of MFE-296 tumours (A and B) Necropsy tumours from different metastatic sites (as indicated with respective organ name), without (A) or with (B) SB-431542 treatment, were stained H&E, and tumours metastasized to different body organs were processed for vimentin immunohistochemistry**.** Organ image scale bar, 1 cm; H&E scale bar, 2 mm; IHC scale bar, 2 mm. Representative images are shown from 2 to 3 tumour sections.

For Supplementary Tables 1-5 see in supplementary Files.
